# Genome sequence of *Ceratocystis huliohia*, a fungal pathogen of the native ‘Ōhi‘a tree in Hawai‘i

**DOI:** 10.1128/mra.00236-26

**Published:** 2026-05-06

**Authors:** Anne Nakamoto, Lisa Keith, Qingyi Yu, Lionel Sugiyama, Xiaohua Wu, Blaine Luiz, MaryAnn Villalun, Jodie Jacobs, Russ Corbett-Detig, Ariana Cisneros, Harrison D. Heath, Cole Shanks, Faith Okamoto, Alexis Abigail Aroma Alburo, Kyle Henricson, Yi Jun Lan, Henry Moore, William Seligmann, Yulia Zybina

**Affiliations:** 1 Department of Biomolecular Engineering, University of California Santa Cruz 8787, Santa Cruz, California, USA; 2 Genomics Institute, University of California Santa Cruz 8787, Santa Cruz, California, USA; 3 Tropical Plant Genetic Resources and Disease Research Unit, Daniel K Inouye U.S. Pacific Basin Agricultural Research Center, Agricultural Research Service, U.S. Department of Agriculture 57524 , Hilo, Hawaii, USA; University of Strathclyde, Glasgow, United Kingdom

**Keywords:** *Ceratocystis huliohia*, Rapid ʻŌhiʻa Death, *Metrosideros polymorpha*, fungal pathogen, long-read genome assembly, Nanopore sequencing, plant pathology, Ascomycota, invasive species, conservation

## Abstract

We present the genome sequence of *Ceratocystis huliohia*, one of two fungal pathogens causing the Rapid ‘Ōhi‘a Death disease of the native ‘ōhi‘a tree in Hawai‘i. This assembly was generated using long-read Nanopore sequencing of *C. huliohia* isolate C25-5 collected on the island of Maui in April 2025.

## ANNOUNCEMENT

Hawai‘i’s native forests are threatened by Rapid ‘Ōhi‘a Death (ROD), a disease of the keystone and culturally significant ‘ōhi‘a tree (*Metrosideros polymorpha*) species (1, 2). ROD was characterized in 2014 and is caused by two novel fungal pathogens, *Certaocystis lukuohia* and *huliohia* (3). Genomic resources for these pathogens remain limited, and although a long-read reference genome is available for *C. lukuohia* (GCF_044167205.1), comparable resources for *C. huliohia* are scarce. Here, we present a long-read genome assembly of *C. huliohia* isolate C25-5 collected in April 2025 at Pu‘u Kukui Elementary School on Maui Island. This resource will facilitate further investigation into the origins of ROD in Hawai‘i.

C25-5 was isolated from discolored wood of an infected tree via carrot baiting, then transfer of ascospores to 10% V8 agar. Taxonomic identity was determined based on morphology and confirmatory qPCR assay (4). We prepared C25-5 for DNA isolation following standard methods for filamentous fungi (5, 6), starting with growth in liquid media (2% malt extract, 0.2% yeast extract) on a circular shaker (150 RPM, 25°C) for 6 days. Mycelium was collected by filtration with miracloth (Millipore, #475855), lyophilized for 2 days, and ground with beads (Geno/Grinder 2010, 1600 RPM, 2 min). We extracted DNA from the ground mycelium using the Wizard HMW DNA Extraction Kit (Promega, A2920) and performed library preparation with the Native Barcoding Kit V14 (Oxford Nanopore Technologies, SQK-NBD114-24). Fragments >3 kb were selected using long-fragment buffer, and no shearing was performed. We sequenced the libraries on a Nanopore MinION Mk1B with an R10 version flow cell (FLO-MIN-114) using MinKNOW v25.05.14 software. Sequencing was stopped after 72 h, and the 1,572,713 raw reads (N50 3.23 kb) basecalled with Dorado v1.1.1 sup model (https://github.com/nanoporetech/dorado) were assembled with hifiasm v0.25.0-r726 ONT mode (7, 8). Because Dorado trims adapters, and hifiasm ONT excludes reads <1 kb or with a *q*-score <10, the reads were not further cleaned. We identified ‘AACCCT’ telomeric repeats with tidk v0.2.65 (9), trimmed low-coverage ends outside of terminal telomeres, and split contigs at internal telomeres to correct misassemblies (telomere_curation.py). Blastn v2.16.0+ (10) of the initial 106 contigs against an available *C. huliohia* mitochondrial genome (MT331822.1) identified one circular contig as the full mitochondrial genome and 89 mitochondrial fragments to exclude. We rotated the mitochondrial genome (PX891014.1) to start at the same position as MT331822.1. Funannotate v1.8.17 (11) was used to mask repeats and annotate genes, and assembly quality and completeness were assessed with QUAST v5.3.0 (12) and BUSCO v6.0.0 (13), respectively. Default parameters were used, except where otherwise specified.

**Fig 1:**
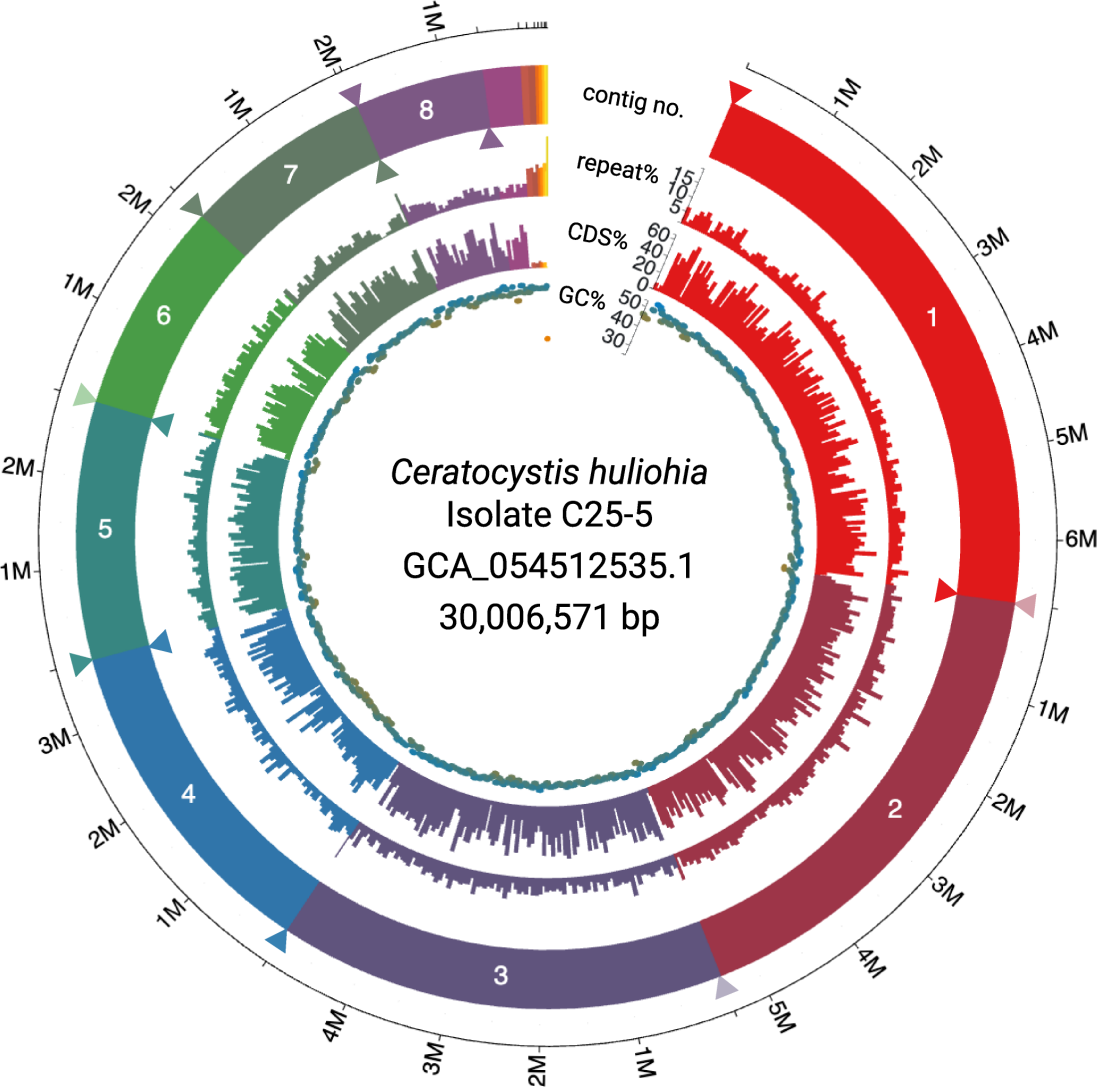
C25-5 genome map. From outermost track inwards: contig size, contig number (for the eight largest) with flanking telomeric repeats indicated by triangles (outer = 5′ end, inner = 3′ end), % repeat content, % CDS content, and % GC content. Plotted using circa.omgenomics.com.

The final C25-5 genome assembly (GCA_054512535.1) had a length of 30.0 Mb and consisted of 16 contigs at 57× coverage, with N50 4.9 Mb, 48.9% guanine-cytosine (GC) content, and 4.8% masked repeats. It demonstrates high completeness, with a BUSCO score indicating 99.7% of fungal orthologs present, and 7,356 predicted genes (Table 1). The majority of bases (97.6%) and coding sequences (98.6%) reside in the eight largest contigs >1 Mb, all with telomeric repeats on at least one end. Of those, five had telomeres on both ends and represent putative chromosomes (Fig. 1). Our assembly appears to agree with the ~7–9 chromosomes expected for *Ceratocystis* species (14); however, further work is required to fully determine *C. huliohia’s* chromosome number.

**TABLE 1:** Isolate, assembly, and annotation summary

Attribute	Value
C25-5 isolate information	
Sample date	11 April 2025
Sample location	Pu‘u Kukui Elementary School, island of Maui
Latitude	20.88
Longitude	−156.51
C25-5 assembly summary statistics	
Total number of contigs	16
Total assembly length	30,006,571 bp (30.0 Mb)
Number of contigs with length >1 Mb	8
Total length of >1 Mb contigs	29,299,846 bp (29.3 Mb)
Percentage of bases in >1 Mb contigs	97.6%
Contig N50	4,906,038 bp (4.9 Mb)
Contig L50	3
Average coverage	57.5×
GC content	48.9%
CDS content	37.2%
Repeat content	4.77% (1,430,314 bp)
BUSCO completeness (fungi_odb10)	99.7%
BUSCO completeness (ascomycota_odb10)	98.2%
BUSCO completeness (sordariomycetes_odb10)	93.5%
C25-5 annotation summary statistics	
Number of genes	7,356 (7,006 mRNA, 350 tRNA)
Average gene length	1,710.6 bp
Number of CDS	17,890
Number of CDS in >1 Mb contigs	17,645 (98.6%)
Average exon length	557.7 bp
Total annotations	35,473
Secretome annotations	558
Transmembrane annotations	1,330
Secondary metabolite biosynthesis gene clusters	11
Biosynthetic enzymes	11
smCOGs	23
CAZYmes	192

## Data Availability

All data associated with this project can be found at NCBI BioProject accession PRJNA1400107. Raw sequencing reads have been deposited in the Sequence Read Archive under accession SRR36741775. The nuclear genome assembly and gene annotations are available under accession GCA_054512535.1, and the mitochondrial genome is available under accession PX891014.1 at GenBank. Data analysis scripts are available at https://github.com/aanakamo/Chuli_C25-5_genome_announcement.
